# Host genetic susceptibility to mycetoma

**DOI:** 10.1371/journal.pntd.0008053

**Published:** 2020-04-30

**Authors:** Rayan S. Ali, Melanie J. Newport, Sahar Mubarak Bakhiet, Muntaser E. Ibrahim, Ahmed Hassan Fahal

**Affiliations:** 1 The Mycetoma Research Centre, University of Khartoum, Khartoum, Sudan; 2 Brighton and Sussex Centre for Global Health Research, Brighton and Sussex Medical School, Brighton, United Kingdom; 3 Institute of Endemic Diseases, University of Khartoum, Khartoum, Sudan; Faculty of Science, Ain Shams University (ASU), EGYPT

## Abstract

Mycetoma is one of the badly neglected tropical diseases, characterised by subcutaneous painless swelling, multiple sinuses, and discharge containing aggregates of the infecting organism known as grains. Risk factors conferring susceptibility to mycetoma include environmental factors and pathogen factors such as virulence and the infecting dose, in addition to host factors such as immunological and genetic predisposition. Epidemiological evidence suggests that host genetic factors may regulate susceptibility to mycetoma and other fungal infections, but they are likely to be complex genetic traits in which multiple genes interact with each other and environmental factors, as well as the pathogen, to cause disease. This paper reviews what is known about genetic predisposition to fungal infections that might be relevant to mycetoma, as well as all studies carried out to explore host genetic susceptibility to mycetoma. Most studies were investigating polymorphisms in candidate genes related to the host immune response. A total of 13 genes had allelic variants found to be associated with mycetoma, and these genes lie in different pathways and systems such as innate and adaptive immune systems, sex hormone biosynthesis, and some genes coding for host enzymes. None of these studies have been replicated. Advances in genomic science and the supporting technology have paved the way for large-scale genome-wide association and next generation sequencing (NGS) studies, underpinning a new strategy to systematically interrogate the genome for variants associated with mycetoma. Dissecting the contribution of host genetic variation to susceptibility to mycetoma will enable the identification of pathways that are potential targets for new treatments for mycetoma and will also enhance the ability to stratify ‘at-risk’ individuals, allowing the possibility of developing preventive and personalised clinical care strategies in the future.

## Background

Mycetoma is a badly neglected tropical disease. It is a chronic granulomatous infectious disease characterised by painless subcutaneous swelling associated with multiple sinuses and discharge that contain aggregates of the infecting organism known as grains [1,2]. The disease is classified according to its causative organisms into actinomycetoma, which is caused by actinomycetes bacteria, and eumycetoma, which is caused by fungi [3]. The suspected route of infection is through traumatic inoculation of environmental microorganisms into the subcutaneous tissue [4,5]. Other mechanisms of transmission (for example, inoculation via insect bites) have not been excluded. Mycetoma has a worldwide distribution, but it is endemic in tropical and subtropical regions in what is known as the ‘mycetoma belt’ between the latitudes of 15°S and 30°N. This belt includes Sudan, Somalia, Senegal, Yemen, India, Mexico, Venezuela, Columbia, and Argentina [6,7]. Sudan seems to be the most highly endemic country for mycetoma worldwide [8]. Mycetoma is seen more frequently amongst impoverished communities in remote rural areas [9]. The majority of mycetoma patients are of low socioeconomic status with little health education. Hence, most of the patients present late with advanced disease, massive deformity, disability, and high morbidity [10,11].

Mycetoma was only recently recognised as a neglected tropical disease in May 2016 by the World Health Organization (WHO) [8,12], and much of the basic information on mycetoma is lacking, including the true incidence, prevalence, and burden of disease; the route of infection; and which risk factors predispose individuals to disease susceptibility. Risk factors that could predispose individuals to mycetoma include environmental factors such as climatic conditions and pathogen factors such as virulence and the infecting dose, in addition to host factors such as immunological status, genetic predisposition, nutritional status, immunosuppression from HIV, coinfections, and use of drugs such as antibiotics or steroids.

The host immune response towards mycetoma-causative organisms has been studied on a limited scale, targeting only a few of the approximately 70 different causative microorganisms of mycetoma. Innate immune responses are a prominent factor in mycetoma because the role of neutrophils in the early defence against mycetoma was demonstrated in previous studies that reported the presence of large numbers of neutrophils in the mycetoma lesion [13,14]. There are 3 host tissue reactions to mycetoma, which include neutrophil adherence and degranulation, resulting in grain disintegration; replacement of neutrophils with macrophages to engulf grain and neutrophil debris; and formation of epithelioid granuloma [14]. Cell-mediated immunity is also required for immunity in mycetoma, with T lymphocytes playing a central role. T helper (Th) type 1 lymphocyte responses provide protective immunity against mycetoma, whilst progression of the disease is linked to Th2 immune response, as previously demonstrated by the significantly higher levels of Th2 cytokines (interleukin [IL]-4, IL-5, IL-6, and IL-10) in mycetoma patients [15–17]. A recent study also pointed a possible role of IL-35 and IL-37 in the pathogenesis of *Madurella mycetomatis*-induced eumycetoma [18]. In 1977, Mahgoub and colleagues suggested that genetic defects in underlying cell-mediated immunity [19] may play a role in the pathogenesis of eumycetoma [16]. Coinfection with schistosomiasis was found to be associated with susceptibility to mycetoma in a small case–control study of 84 subjects in Sudan [16]. The authors hypothesised that the prolonged Th2 response, induced by schistosomiasis, predisposed individuals to develop mycetoma [16]. The humoral arm of the acquired immunity against mycetoma was evaluated by Wethered and his colleagues using an enzyme-linked immunosorbent assay (ELISA) to measure immunoglobulin levels in serum collected from cases and controls [20]. High levels of immunoglobulin M (IgM) were seen in most patients with mycetoma due to *M*. *mycetomatis*, whereas low levels of specific IgG were detected in some patients. Additionally, IgM, IgG, and complement factors were identified on the surface of the grains taken from actinomycetoma lesions caused by *Streptomyces somaliensis* [15].

In this review, genetic predisposition to fungal infections resembling mycetoma was reviewed to give clues about similar genetic variations that predispose individuals to mycetoma. Additionally, all studies that were previously done to explore host genetic susceptibility to mycetoma were also included.

## Methods

An extensive literature review was conducted using the electronic databases PubMed/MEDLINE and Google Scholar. The search included combinations of the following key words: ‘mycetoma’, ‘polymorphisms’, and ‘genetic susceptibility’ for reviewing articles regarding susceptibility to mycetoma. For genetic susceptibility to fungal infections, the key words ‘fungal’, ‘infection’, ‘mycoses’, and ‘genetic susceptibility’ were used as search terms. All articles identified through the 2 electronic databases were analysed to ensure they were within the scope of this review. Only papers written in English were included, and year of publication was not restricted.

## Host genetic susceptibility to mycetoma and other fungal infections

Whilst there is a clear link to low socioeconomic development, only a proportion of people exposed to mycetoma-causing organisms develop clinical disease, despite a shared environment and the ubiquitous environmental presence of the pathogens in endemic areas. This observation raises the hypothesis that host genetic factors have a role in determining the outcome of infection.

Three previous studies used ELISA to demonstrate the presence of antibodies against mycetoma-causative agents in the sera of unaffected residents in endemic areas, indicating exposure to the pathogens without development of disease [21–23].

Other evidence suggesting a genetic predisposition to mycetoma includes familial clustering of mycetoma patients, first observed by Al Dawi and her colleagues in 2013 in a hospital-based study at the Mycetoma Research Centre (MRC) that included 53 eumycetoma patients and 31 healthy controls [24]. This study showed that more than 62% of eumycetoma patients had at least one family member affected by the disease. A community-based study in 2014 also suggested a role for genetic inheritance, with more than half of patients (52%) having a family member with the disease [10]. In 2015, a comprehensive study from the MRC reported a family history of mycetoma in 12% of 6,792 patients seen during the period 1991–2014 [25]. No studies have been undertaken to date to estimate heritability, investigate the disease’s mode of inheritance, or confirm the genetic component of susceptibility towards mycetoma based on the previously observed familial clustering. It is important to note that families share their environment as well as their genes, but in communities affected by mycetoma, families with multiple cases live in close proximity to families who have no cases, suggesting gene–environment interactions are indeed important.

Finally, there is a good evidence in other fungal infections that host genetic factors determine susceptibility to disease [26]. Examples include phaeohyphomycosis, chromoblastomycosis, candidiasis, and aspergillosis, in which a complex range of clinical phenotypes is also observed.

In summary, there is some evidence suggesting host genetic factors as regulators of susceptibility to mycetoma and other fungal infections, but they are likely to be complex genetic traits in which multiple genes interact with each other and environmental factors, as well as the pathogen, to cause disease. It is therefore challenging to identify the genes involved. One approach that has successfully identified genes associated with infectious diseases is the characterisation of rare monogenic disorders that predispose individuals to infection. Whilst rare, investigation of such families offers insights into critical immune response pathways that can be further investigated at the population level. Examples here include mutations in genes within the interferon (IFN)-gamma pathway that predispose individuals to mycobacterial infection [27] or genes encoding terminal complement pathway components that predispose individuals to meningococcal infection [28]. This approach has also been applied to fungal infections.

### Monogenic disorders and fungal infections

Single-gene defects that lead to primary immunodeficiencies (PIDs) have helped to understand better the immunological defects that increase susceptibility to fungal infections. An example of an autosomal recessive PID is human caspase recruitment domain-containing protein 9 (CARD9) deficiency, which is caused by biallelic mutations in the gene *CARD9* [29]. *CARD9* encodes for an adaptor protein downstream from C-type lectin receptors (CLRs), which are pathogen recognition receptors (PRRs), like the Toll-like receptors (TLRs), that have a major role in fungal recognition [30]. In humans, CLRs (for example, dectin-1, dectin-2, and the macrophage inducible Ca 2+-dependent lectin receptor MINCLE) that can recognise fungal pathogen-associated molecular patterns (PAMPs) and their adaptor molecule CARD9 have a critical role in antifungal host defence, and several studies highlighted an enhanced fungus-specific infection susceptibility as a consequence of mutations or deletions in CLRs or *CARD9* [30,31]. For example, autosomal recessive mutations in *CARD9* cause significant defects in the Th17 response because of decreased proportions of circulating IL17^+^ T lymphocytes [32]; diminished production of IL1β and IL6, which are essential for priming Th17 cell differentiation; and impaired neutrophil killing [33]. Interestingly, 2 compound heterozygous mutations and 1 homozygous frameshift mutation in *CARD9* were also found in patients with phaeohyphomycosis, which is a subcutaneous infection similar to mycetoma [34]. Phaeohyphomycosis is caused by several microorganisms, including *Phialophora verrucose*, which is one of the mycetoma-causative organisms [35]. The identified mutations did not affect *CARD9* expression but resulted in lack of the wild-type mature CARD9 protein, which consequently decreased TH17 cell proportions and cytokine production and impaired patients’ immune responses. Recently, Queiroz-Telles and colleagues successfully used hematopoietic stem cell transplantation (HSCT) to treat 2 unrelated patients from Brazil and Morocco with deep/invasive dermatophytosis caused by CARD9 deficiency [36]. Both patients stopped antifungal therapy after achieving complete clinical remission, suggesting that the pathogenesis of fungal infections in these patients was mainly due to the disruption of leukocyte-mediated CARD9 immunity. *P*. *verrucosa* causes another disease that resembles mycetoma known as chromoblastomycosis (CBM). Familial inheritance to CBM was suggested by Pérez-Blanco and colleagues, with a 3.5 times higher risk of developing CBM amongst members of common ancestry in an endemic state in Venezuela [37]. In Brazil, Tsuneto and colleagues found in a study involving 32 CBM patients and 77 healthy matched controls that the human leukocyte antigen (HLA)-A29 was significantly associated with susceptibility to the disease (*P* value = 0.03) [38]. The relative risk of individuals with HLA-A29 antigen was estimated to be 10-fold higher than those lacking the antigen.

Another known PID is chronic granulomatous disease (CGD), which results from defects in genes encoding the NADPH oxidase subunits (*CYBA* and *CYBB*, encoding the cytochrome B-245 alpha and beta chains, respectively; and *NCF-1*, *NCF-2*, and *NCF-4*, encoding neutrophil cytosolic factors 1, 2 and 4), which together are critical for the generation of superoxide within phagocytes [39]. In 65% of CGD cases, an X-linked recessive pattern of inheritance is found because of mutations in *CYBB* encoding subunit gp91^phox^. The remaining 35% of cases are autosomal recessive resulting from mutations in *CYBA*-, *NCF-1*–, *NCF-2*–, and *NCF-4*–encoding subunits p22^phox^, p47^phox^, p67^phox^, and p40^phox^, respectively. The mutations in the genes encoding NADPH oxidase subunits include deletions, frameshifts, and nonsense and missense mutations [40]. This defect in CGD phagocytes increases susceptibility to fungal infections such as aspergillosis (in one-third of all CGD cases) [39] and candidiasis [41]. The important function of neutrophils in immunity against mycetoma has been demonstrated [1,42], suggesting that investigating similar mutations that potentially lead to impaired oxygen-dependent fungicidal activity in eumycetoma may be fruitful.

Chronic mucocutaneous candidiasis (CMC) is a monogenetic immunodeficiency of cell-mediated immunity that develops as a consequence of defects in IL-17 and IL-22 immunity required for defence against fungal infections [43]. CMC is transmitted with autosomal dominant or recessive inheritance patterns and affects the skin, nails, and mucous membranes of affected patients [41]. The autosomal dominant pattern of CMC is mainly caused by gain-of-function missense mutations in the CC-domain of *STAT1* (Signal Transducer And Activator Of Transcription 1), which encodes a critical transcription factor downstream from IFN-α/β and IFN-γ signalling. These mutations led to defective Th1 and Th17 lymphocyte responses and reduced IL-17, IL-22, and IFN-γ production, explaining the increased susceptibility to fungal infection [44]. In addition, mutations in *IL17RA* and *IL17F* have been associated with autosomal dominant CMC, leading to impaired IL-17 immunity [44]. Autosomal recessive CMC results from a rare condition, autoimmune polyendocrinopathy candidiasis ectodermal dystrophy (APECED), which in turn results from mutations in the autoimmune regulator (*AIRE*) gene [39]. *AIRE* encodes a transcriptional regulator expressed by medullary thymic epithelial cells to regulate the expression of peripheral tissue-specific self-antigens and promote central tolerance via deletion of self-reactive T cells [45]. Loss-of-function mutations in *AIRE* lead to the production of neutralising autoantibodies against important cytokines with antifungal properties such as IL-17E, IL-17F, and IL-22 [46].

Since deficiency in cell-mediated immunity in mycetoma patients was previously suggested by Mahgoub and colleagues [19], the presence of similar mutations in eumycetoma should be studied. The importance of IL-17 immunity against *Aspergillus fumigatus* and *Fusarium oxysporum*, which are both causative agents of mycetoma [47,48], was demonstrated by Taylor and colleagues in murine models of fungal keratitis [49]. Both IL-17–producing neutrophils and Th17 cells conferred protective immunity against fungal hyphae and regulated the severity of corneal disease. In humans, Siddig and colleagues demonstrated the expression of IL-17A in the granuloma of different mycetoma-causative agents, reaffirming the importance of Th17 immunity against mycetoma infection [50].

### Polygenic disorders and fungal infections

Although very informative, single-gene mutations leading to fungal infections are relatively rare in the general population, in which complex inheritance of traits is more likely. Recent advances in genome interrogation technologies have enabled researchers to detect genetic variations that predispose susceptibility to fungal infections and the interplay between innate and adaptive immune cells in addition to other effector cells that together constitute the host immune response to fungal invasion [41,51]. For example, genetic susceptibility to candidiasis was investigated using genome-wide association studies (GWASs), which revealed a 19.4-fold increased risk in individuals with 2 or more single-nucleotide polymorphisms (SNPs) in *LFA-3* (lymphocyte function-associated antigen 3), *LCE4A* (late cornified envelope 4A), and *TAGAP* (T cell activation RhoGTPase activating protein) loci [52].

Genetic susceptibility to aspergillosis is also polygenic [53]. *Aspergillus* spp. are seldom pathogenic in immunocompetent hosts because of innate immune responses mediated mainly through alveolar macrophages and neutrophils [54]. Most cases of aspergillosis occur in the context of down-regulation of the immune system by immunosuppressive therapies. Susceptibility to different forms of aspergillosis, including invasive allergic (IA) and chronic noninvasive syndromes, has been shown to be associated with polymorphic variants in *TLR1*, *TLR6*, *TLR4*, *TLR9*, and *TLR3* [55–57]. Variants in the chemokine (C-X-C motif) ligand 10 (*CXCL10*), an inflammatory mediator that stimulates the directional migration of Th1 in addition to increasing T cell adhesion to endothelium [58], have been associated with invasive *A*. *fumigatus* infection [59]. In this study, the presence of a haplotype in *CXCL10* (rs1554013 [+11101 **C**/T], rs3921 [+1642 C/**G**], and rs4257674 [−1101 **A**/G]) was associated with the inability of immature dendritic cells (iDCs) to express *CXCL10* in patients with IA after allogeneic HSCT, which led to an increased risk of developing IA. Susceptibility to IA has also been linked to deficiencies in PRRs with opsonic activity such as mannose-binding lectin (*MBL*) and long pentraxin 3 (*PTX3*), which mediates fungal uptake and killing by phagocytes [30,60,61]. Another molecule with an opsonic activity that has been associated with susceptibility to IA in allogeneic HSCT patients is plasminogen (*PLG*) [62]. Using a novel method of candidate gene polymorphisms identification, Zaas and colleagues used computational genetic mapping analysis of suppressed murine model survival data to eventually identify an SNP in human *PLG* that increased the risk for IA in HSCT recipients.

### Genetic susceptibility to mycetoma

Most of the studies done on host susceptibility to mycetoma focused on polymorphisms in candidate genes related to the host immune response.

#### Polymorphisms in innate immunity genes

Because of the significance of innate immunity in host defence, genetic variations in the genes of this system could have a major impact on immune responses to mycetoma-causative organisms. Thus, the first evidence of host genetic susceptibility to mycetoma was shown in 2007 by van de Sande and her colleagues when they reported associations between polymorphisms in genes involved in innate immunity and susceptibility to mycetoma [42]. Studies initially focused on neutrophil function given its importance in early defence against mycetoma [13,14]. Studies were undertaken on 11 SNPs in 8 genes: complement receptor 1 (*CR1*), *MBL*, tumour necrosis factor α (*TNFα*), macrophage chemoattractant protein-1 (*MCP-1*), IL 8 (*CXCL8*), C-X-C motif chemokine receptor 2 (*CXCR2*), nitric oxide synthase 2 (*NOS2*), and thrombospondin-4 (*TSP-4*). Five SNPs in *CR1*, *CXCL8*, its receptor *CXCR2*, *TSP-4*, and *NOS2* had significant differences in allele distribution between 125 Sudanese mycetoma patients and 140 matched controls. Furthermore, an SNP in *NOS2* was associated with lesion size. The SI1 and McC^a^ alleles of CR1 SNPs rs17047661 and rs17047660, respectively, had higher distribution in mycetoma patients than in matched endemic controls, and the authors speculated that these polymorphisms could result in conformational changes in the receptor that eventually might lead to a defect in the efficacy of *M*. *mycetomatis* phagocytosis. CXCL8 and TSP-4 are chemoattractants for neutrophils. The alleles −251A allele in *CXCL8* and the 29929C allele in *TSP4*, in addition to the +785C allele in *CXCR2* that encodes for the CXCL8 receptor, were associated with mycetoma in this study. The final polymorphism for which there were differences in allele frequency between mycetoma patients and healthy controls was found in *NOS2*, encoding nitric oxide synthase, which produces nitric oxide (NO), which mediates tumoricidal and bactericidal actions [63]. The G954C allele results in a 7-fold higher NOS activity compared to the wild type, which may explain why it was found more amongst the healthy endemic controls [64]. Higher production of CXCL8 and lower NO production in mycetoma patients were both highlighted as risk factors for developing mycetoma owing to impaired wound healing, which was previously demonstrated in mice [65,66].

The absence of significant differences in allele frequencies between cases and endemic matched controls in polymorphisms in the other 6 genes might be due to investigating a limited number of variants in addition to the small number of enrolled subjects, resulting in a lack of statistical power. Thus, exploring other variants in these genes in addition to other genes involved in neutrophil function in a larger cohort could reveal additional variants with functional impact in susceptibility to mycetoma.

#### Polymorphisms in acquired immunity genes

Several HLA alleles (both class I and class II) have been associated with susceptibility or resistance to the development of infectious diseases [67–69]. Based on this, Al Dawi and colleagues analysed *HLA-DRB1* and *HLA-DQB1* allele frequencies amongst confirmed eumycetoma patients compared with matched controls [24]. Of the *HLA-DRB1* alleles, the HLA-DRB1*13 allele showed significant association with eumycetoma infection (*P* = 0.044, odds ratio [OR] = 2.629). Interestingly, the HLA-DRB1*02 allele had a high frequency in the control group (9.8%, *P* = 0.047) whilst it was absent in the eumycetoma patients, suggesting a protective role against eumycetoma. For *HLA-DQB1* alleles, the HLA-DQB1*5 allele showed a significantly higher frequency in mycetoma patients than in healthy controls (*P* = 0.029, OR = 3.471), indicating a possible association between this allele and the development of clinical mycetoma. This study included only 84 subjects (53 eumycetoma patients and 31 healthy matched controls) who were admitted to the MRC. Therefore, further studies are required on larger sample sizes and amongst communities living in mycetoma-endemic areas.

A number of studies have identified association between variants in cytokine genes involved in acquired immune responses and mycetoma. Owing to the important roles of C-C chemokine ligand 5 (CCL5) in attracting T cells, dendritic cells, eosinophils, and natural killer (NK) cells [70] and IL-10, which is one of the Th 2 cytokines [71], Mhmoud and colleagues studied the role of 3 functional SNPs in *CCL5* and 2 SNPs in *IL-10* in mycetoma granuloma formation [17]. Allele frequencies for 2 SNPs in *CCL5* (rs2280788 and rs280789) and 1 SNP in *IL-10* (rs280789) were significantly different between 149 mycetoma patients and 206 matched controls and were linked to elevated serum levels of both CCL5 and IL-10 in mycetoma patients. The 2 SNP alleles in *CCL5* that were significantly associated with mycetoma had opposite functional effects: the G-allele at SNP rs2280788 results in a higher *CCL5* transcription, whilst the C-allele in the other SNP, rs280789, is associated with diminished transcription of *CCL5*. Consequently, the exact role of CCL5 in mycetoma granuloma formation remains to be elucidated.

#### Polymorphisms in host enzyme genes

Two studies involving host enzymes that are part of the immune system have been reported. The first, reported in 2014 by Geneugelijk and colleagues [72], investigated the role of matrix metalloproteinases-2 (MMP-2), matrix metalloproteinases-9 (MMP-9), and tissue inhibitor of metalloproteinases (TIMP) in the formation of fungal grains in *M*. *mycetomatis* eumycetoma patients. These enzymes are thought to be involved in the deposition of collagen around the grains to encapsulate them within the granulomas [72]. It is believed that excessive collagen formation may contribute to treatment failure in mycetoma patients. The study included 3 parts: (1) detection of MMP-2 and MMP-9 locally around fungal grains using immunohistochemical staining of 8 tissue sections taken from *M*. *mycetomatis* eumycetoma patients, (2) measuring absolute serum levels of MMP-2 and MMP-9 in the sera of 36 *M*. *mycetomatis*-infected patients and 36 healthy controls using ELISA, and (3) genotyping functional SNPs in the promoter regions of *MMP-2* (rs243865), *MMP-9* (rs3918242), and *TIMP-1* (rs4898) using genomic DNA from 125 Sudanese *M*. *mycetomatis* eumycetoma patients and 103 healthy endemic controls. Active MMP-9 was only found in the sera of 36% of eumycetoma patients, yet that cannot be attributed solely to mycetoma infection because no data about coinfection were recorded during the sample collection. No genetic differences were found between patients and healthy controls for *MMP-2* and *MMP-9*; however, there was a higher frequency of the T allele of an SNP in *TIMP-1* (372T/C) in 77 male eumycetoma patients compared to 44 healthy male controls. This T allele is associated with a lower TIMP-1 protein expression in inflammatory bowel disease, and the authors suggested that it could explain the previously reported male predominance in mycetoma [73].

In 2015, a second study investigated the presence of chitin in the cell wall of *M*. *mycetomatis*. Chitin triggers the host innate immune response and the release of chitin-degrading enzymes such as chitotriosidase (*CHIT1*) and acidic mammalian chitinase (AMCase, also known as *CHIA*) [74]. Four polymorphisms in *CHIT1* and *CHIA* were studied in 112 eumycetoma patients and 103 matched controls that were from the same cohort used for the metalloproteinases study. Though both *CHIA* and *CHIT1* were expressed in mycetoma lesions, a 24-bp insertion in *CHIT1* resulting in impaired enzyme activity [75,76] was found to be significantly associated with eumycetoma. Because both chitotriosidase and AMCase were proven to exist in mycetoma lesions, further investigation to detect polymorphisms affecting the activity of these enzymes is still needed with a larger sample size.

#### Polymorphisms in sex hormone biosynthesis genes

A male predominance of mycetoma has been described in several studies [7,9,11,72], suggesting a possible role of hormonal influence in mycetoma susceptibility. Based on this observation, a study was carried out in 2015 on 125 eumycetoma patients and 103 matched controls (the same metalloproteinases study group) to investigate changes in hormonal levels that could result from polymorphisms within genes encoding enzymes essential for sex hormone biosynthesis [77]. The selected polymorphisms were rs4680 in Catechol-O-methyltransferase (*COMT*), rs1056836 in Cytochrome p450 subfamily 1B1 (*CYP1B1*), rs743572 in Cytochrome p450 subfamily 17 (*CYP17*), rs700518 in cytochrome p450 subfamily 19 (*CYP19*), and rs6203 in hydroxysteroid dehydrogenase 3B (*HSD3B*). Only alleles of the rs4680 SNP in *COMT*, which is quite common in African populations according to the Genome Aggregation Database (gnomAD) [78] and has been linked to neuropsychological disorders, and rs700518 in *CYP19* had significant differences in distribution amongst mycetoma patients compared to healthy endemic controls.

In summary, a total of 13 genes (Table 1) have allelic variants found to be associated with mycetoma, and these genes lie in different pathways and systems. All the studies mentioned above were focused on a predefined set of polymorphisms in candidate genes, selected for their possible role in immunity to mycetoma in relatively small sample sizes. No systematic genome-wide studies have been reported to date.

**Table 1:** A summary of genes with allelic variants that are significantly associated with mycetoma.

Gene	SNP	Rs-Number	*P* Value	Reference
***CCL5***	−28C/G	rs2280788	<0.0001	[17]
	1648044C>T*	rs280789	<0.0001	[17]
***CHIT1***	24-bp insertion	rs3831317	0.004	[74]
*COMT*	19951271G>A*	rs4680	0.006	[77]
***CR1***	207782889A>G*	rs17047661	0.039	[42]
	207782856A>G*	rs17047660	0.001	[42]
***CXCL8***	−251T/A	rs4073	0.008	[42]
*CXCR2*	219000310C>T*	rs2230054	0.037	[42]
***CYP19***	51529112T>C*	rs700518	0.004	[77]
*HLA-DRB1*	HLA-DRB1*13	N/A	0.044	[24]
	HLADRB1*02	N/A	0.047	[24]
*HLA-DQB1*	HLA-DQB1*5	N/A	0.029	[24]
***IL-10***	−819T/C	rs1800871	0.0005	[17]
*NOS2*	−954 G>C*	rs1800482	0.0006	[42]
***TIMP-1***	372T/C	rs4898	0.0004 (males); 0.53 (females)	[72]
*TSP4*	79361265G>C*	rs1866389	0.030	[42]

* GRCh37 human reference genome.

**Abbreviations:** *CCL5*, C-C chemokine ligand 5; *CHIT1*, chitotriosidase; *COMT*, catechol-O-methyltransferase; *CR1*, complement receptor 1; *CXCL8*, C-X-C chemokine ligand 8; *CXCR2*, C-X-C motif chemokine receptor 2; *CYP19*, cytochrome p450 family 19; *HLA*, human leukocyte antigen; *IL-10*, interleukin 10; *NOS2*, nitric oxide synthase 2; N/A, not applicable; *TIMP-1*, tissue inhibitor of metalloproteinases 1; *TSP4*, thrombospondin-4.

## Concluding remarks and future perspectives

The studies described above suggest there is a role for the host’s underlying genetic profile in determining susceptibility to mycetoma. However, the studies are generally small and take a candidate gene approach, and none of them have been replicated. Advances in genomic science and the supporting technology have paved the way for large-scale genome-wide association and next generation sequencing (NGS) studies that can underpin a new strategy to systematically interrogate the genome for variants associated with mycetoma. GWASs are used to detect common variants, whilst NGS technologies such as whole-exome sequencing (WES), in which the exons across the whole genomes of cases and controls are sequenced, are used to identify the rare variants with functional impact on disease pathogenesis [79]. Since several genomes for the most common causative organisms, including *Nocardia brasiliensis* [80], *S*. *somaliensis* [81], *Actinomadura madurae* [82], *Nigrograna mackinnonii* [83], and *M*. *mycetomatis* [84] are now publicly available, parallel analysis of pathogen and host genomes could give valuable insights into host–pathogen interactions in mycetoma [1] and would eventually aid in devising better strategies for risk assessment, treatment, and prognosis for mycetoma patients.

It is essential to include large cohorts to avoid the multiple challenges of genetic association studies, including population stratification, genetic heterogeneity, and insufficient replication power [85,86]. Additionally, genotype–phenotype correlation analyses are mandatory to correlate the relative contribution of genetic findings to the specific clinical outcomes.

Dissecting the contribution that host genetic variation has on susceptibility to mycetoma will enable the identification of pathways that are potential targets for new treatments for mycetoma and will also enhance our ability to stratify ‘at-risk’ individuals, allowing the potential to develop preventive and personalised clinical care strategies in the future.

**Key learning points**

1. Current evidence suggests a role for host genetic factors in regulating susceptibility to mycetoma.
2. Mycetoma is likely to be a complex genetic trait in which multiple genes interact with each other and environmental factors, as well as the pathogen, to cause the disease.
3. Reviewing genetic susceptibility to other morphologically related fungal infections can help to identify candidate genes involved in susceptibility to mycetoma.
4. A total of 13 genes had allelic variants found to be associated with mycetoma.
5. All the reviewed studies on genetic susceptibility to mycetoma took a candidate gene approach, and none have been replicated.
6. There is a need for a new strategy to systematically interrogate the genome for variants associated with mycetoma.

**Top 5 papers**

1. van de Sande WWJ, Fahal A, Verbrugh H, van Belkum A. Polymorphisms in Genes Involved in Innate Immunity Predispose Toward Mycetoma Susceptibility. J Immunol. 2007;179: 3065–3074.
2. Verwer PEB, Notenboom CC, Eadie K, Fahal AH, Verbrugh HA, van de Sande WWJ. A Polymorphism in the Chitotriosidase Gene Associated with Risk of Mycetoma Due to Madurella mycetomatis Mycetoma–A Retrospective Study. PLoS Negl Trop Dis. 2015;9: e0004061.
3. van de Sande WW, Fahal A, Tavakol M, van Belkum A. Polymorphisms in catechol-O-methyltransferase and cytochrome p450 subfamily 19 genes predispose towards Madurella mycetomatis- induced mycetoma susceptibility. Med Mycol 2010; 48: 959–968.
4. Mhmoud NA, Fahal AH, van de Sande WWJ. The association between the interleukin-10 cytokine and CC chemokine ligand 5 polymorphisms and mycetoma granuloma formation. Med Mycol. 2013;(5): 527–33.
5. Al Dawi AF, Mustafa MI, Fahal AH, EL Hassan AM, Bakhiet S, Magoub ES, et al. The association of HLA-DRB1 and HLA-DQB1 alleles and the occurrence of eumycetoma in Sudanese patients. Khartoum Med J. 2013;6: 923–929.

## References

[1] van Belkum A, Fahal A, van de Sande WW. Mycetoma caused by Madurella mycetomatis: a completely neglected medico-social dilemma. Adv Exp Med Biol. 2013;764: 179–89. 10.1007/978-1-4614-4726-9_15 23654067

[2] Lichon V, Khachemoune A. Mycetoma: a review. American Journal of Clinical Dermatology. 2006; 315–321. 10.2165/00128071-200607050-00005 17007542

[3] Nenoff P, van de Sande WW, Fahal AH, Reinel D, Schöfer H. Eumycetoma and actinomycetoma—An update on causative agents, epidemiology, pathogenesis, diagnostics and therapy. J Eur Acad Dermatol Venereol. 2015;29(10): 1873–83. 10.1111/jdv.13008 25726758

[4] de Hoog GS, Ahmed SA, Najafzadeh MJ, Sutton DA, Keisari MS, Fahal AH, et al Phylogenetic Findings Suggest Possible New Habitat and Routes of Infection of Human Eumyctoma. PLoS Negl Trop Dis. 2013;7(5): e2229 10.1371/journal.pntd.0002229 23696914 PMC3656121

[5] Samy AM, van de Sande WWJ, Fahal AH, Peterson AT. Mapping the potential risk of mycetoma infection in Sudan and South Sudan using ecological niche modeling. PLoS Negl Trop Dis. 2014;8: e3250 10.1371/journal.pntd.0003250 25330098 PMC4199553

[6] WHO. Mycetoma. World Health Organization [Internet]. 2016 [cited 2019 Mar 21]. Available from: https://www.who.int/buruli/mycetoma/en/

[7] Fahal AH. Mycetoma: A thorn in the flesh. Trans R Soc Trop Med Hyg. 2004;98: 3–11. 10.1016/s0035-9203(03)00009-9 14702833

[8] Burki TK. Starting from scratch-the unique neglect of mycetoma. Lancet Infect Dis. 2016;16(9): 1011–1012. 10.1016/S1473-3099(16)30283-3 27684346

[9] Queiroz-Telles F, Fahal AH, Falci DR, Caceres DH, Chiller T, Pasqualotto AC. Neglected endemic mycoses. Lancet Infect Dis. 2017 11;17(11): e367–e377. 10.1016/S1473-3099(17)30306-7 28774696

[10] Fahal A, Mahgoub ES, EL Hassan AM, Abdel-Rahman ME, Alshambaty Y, Hashim A, et al A New Model for Management of Mycetoma in the Sudan. PLoS Negl Trop Dis. 2014;8(10): e3271 10.1371/journal.pntd.0003271 25356640 PMC4214669

[11] Zijlstra EE, van de Sande WWJ, Welsh O, Mahgoub ES heikh, Goodfellow M, Fahal AH. Mycetoma: a unique neglected tropical disease. Lancet Infect Dis. 2016;16: 100–112. 10.1016/S1473-3099(15)00359-X 26738840

[12] Ahmed SA, Van De Sande WWJ, Desnos-Ollivier M, Fahal AH, Mhmoud NA, De Hoog GS. Application of isothermal amplification techniques for identification of madurella mycetomatis, the prevalent agent of human mycetoma. J Clin Microbiol. 2015;53: 3280–3285. 10.1128/JCM.01544-15 26246484 PMC4572522

[13] EL Hassan AM, Fahal AH, EL Hag IA, Khalil EAG. The pathology of mycetoma: light microscopic and ultrastructural features. Sudan Med J. 1994;23–45.

[14] Fahal AH, el Toum EA, el Hassan AM, Mahgoub ES, Gumaa SA. The host tissue reaction to Madurella mycetomatis: new classification. J Med Vet Mycol 1995;33: 15–17. 7650573

[15] El Hassan AM, Fahal AH, Ahmed AO, Ismail A, Veress B. The immunopathology of actinomycetoma lesions caused by Streptomyces somaliensis. Trans R Soc Trop Med Hyg. 2001;95(1): 89–92. 10.1016/s0035-9203(01)90346-3 11280076

[16] Nasr A, Abushouk A, Hamza A et al Th-1, Th-2 Cytokines profile among Madurella mycetomatis eumycetoma patients. PLoS Negl Trop Dis. 2016;10: e0004862 10.1371/journal.pntd.0004862 27434108 PMC4951069

[17] Mhmoud NA, Fahal AH, van de Sande WWJ. The association between the interleukin-10 cytokine and CC chemokine ligand 5 polymorphisms and mycetoma granuloma formation. Med Mycol. 2013;(5): 527–33. 10.3109/13693786.2012.745201 23210681

[18] Abushouk A, Nasr A, Masuadi E, Allam G, Siddig EE, Fahal AH. The Role of Interleukin-1 cytokine family (IL-1β, IL-37) and interleukin-12 cytokine family (IL-12, IL-35) in eumycetoma infection pathogenesis. PLoS Negl Trop Dis. 2019;13: e0007098 10.1371/journal.pntd.0007098 30946748 PMC6483278

[19] Mahgoub ES, Gumaa SA, El Hassan AM. Immunological status of mycetoma patients. Bull Soc Pathol Exot Filiales. 1977;70(1): 48–54. 579331

[20] Wethered DB, Markey MA, Hay RJ, Mahgoub ES, Gumaa SA. Humoral immune responses to mycetoma organisms: characterization of specific antibodies by the use of enzyme-linked immunosorbent assay and immunoblotting. Trans R Soc Trop Med Hyg. 1988;82: 918–23. 10.1016/0035-9203(88)90042-9 2476875

[21] Mclaren ML, Mahgoub ES, Georgakopoulos E. Preliminary investigation on the use of the enzyme linked immunosorbent assay (ELISA) in the serodiagnosis of mycetoma. Med Mycol. 1978;16: 225–228. 10.1080/00362177885380301 360443

[22] Taha A. A serological survey of antibodies to Streptomyces somaliensis and Actinomadura madurae in the Sudan using enzyme linked immunosorbent assay (ELISA). Trans R Soc Trop Med Hyg. 1983;77: 49–50. 10.1016/0035-9203(83)90011-1 6679364

[23] van de Sande WW, Janse DJ, Hira V, et al Translationally controlled tumor protein from Madurella mycetomatis, a marker for tumorous mycetoma progression. J Immunol 2006;177: 1997–2005. 10.4049/jimmunol.177.3.1997 16849514

[24] Al Dawi AF, Mustafa MI, Fahal AH, EL Hassan AM, Bakhiet S, Magoub ES, et al The association of HLA-DRB1 and HLA-DQB1 alleles and the occurrence of eumycetoma in Sudanese patients. Khartoum Med J. 2013;6: 923–929.

[25] Fahal A, Mahgoub el S, El Hassan AM et al Mycetoma in the Sudan: an update from the Mycetoma Research Centre, University of Khartoum, Sudan. PLoS Negl Trop Dis. 2015;9: e0003679 10.1371/journal.pntd.0003679 25816316 PMC4376889

[26] Gow NAR, Netea MG. Medical mycology and fungal immunology: new research perspectives addressing a major world health challenge. Philos Trans R Soc B Biol Sci. 2016;371: 20150462. 10.1098/rstb.2015.0462 PMC5095541 28080988

[27] Newport MJ, Huxley CM, Huston S, Hawrylowicz CM, Oostra BA, Williamson R, et al A Mutation in the Interferon-γ –Receptor Gene and Susceptibility to Mycobacterial Infection. N Engl J Med. 1996;335: 1941–1949. 10.1056/NEJM199612263352602 8960473

[28] Figueroa JE, Densen P. Infectious diseases associated with complement deficiencies. Clin Microbiol Rev. 1991;4: 359–95. 10.1128/cmr.4.3.359 1889047 PMC358203

[29] Drummond RA, Lionakis MS. Mechanistic Insights into the Role of C-Type Lectin Receptor/CARD9 Signaling in Human Antifungal Immunity. Front Cell Infect Microbiol. 2016;6:39 10.3389/fcimb.2016.00039 27092298 PMC4820464

[30] Lionakis MS, Levitz SM. Host Control of Fungal Infections: Lessons from Basic Studies and Human Cohorts. Annu Rev Immunol. 2018;36: 157–191. Epub 2017 Dec 13. 10.1146/annurev-immunol-042617-053318 29237128

[31] Gross O, Gewies A, Finger K, Schäfer M, Sparwasser T, Peschel C, et al Card9 controls a non-TLR signalling pathway for innate anti-fungal immunity. Nature. 2006;442: 651–656. 10.1038/nature04926 16862125

[32] Glocker E-O, Hennigs A, Nabavi M, Schäffer AA, Woellner C, Salzer U, et al A Homozygous CARD9 Mutation in a Family with Susceptibility to Fungal Infections. N Engl J Med. 2009;361: 1727–1735. 10.1056/NEJMoa0810719 19864672 PMC2793117

[33] Drewniak A, Gazendam RP, Tool ATJ, van Houdt M, Jansen MH, van Hamme JL, et al Invasive fungal infection and impaired neutrophil killing in human CARD9 deficiency. Blood. 2013;121: 2385–2392. 10.1182/blood-2012-08-450551 23335372

[34] Wang X, Wang W, Lin Z, Wang X, Li T, Yu J, et al CARD9 mutations linked to subcutaneous phaeohyphomycosis and T H17 cell deficiencies. J Allergy Clin Immunol. 2014;133(3): 905–908. 10.1016/j.jaci.2013.09.033 24231284

[35] Turiansky GW, Benson PM, Sperling LC, Sau P, Salkin IF, McGinnis MR, et al Phialophora verrucosa: A new cause of mycetoma. J Am Acad Dermatol. 1995;32: 311–315. 10.1016/0190-9622(95)90393-3 7829731

[36] Queiroz-Telles F, Mercier T, Maertens J, Sola CBS, Bonfim C, Lortholary O, et al Successful Allogenic Stem Cell Transplantation in Patients with Inherited CARD9 Deficiency. J Clin Immunol. Springer New York LLC; 2019;39: 462–469. 10.1007/s10875-019-00662-z 31222666

[37] Pérez-Blanco M, Hernández Valles R, García-Humbría L, Yegres F. Chromoblastomycosis in children and adolescents in the endemic area of the Falcón State, Venezuela. Med Mycol. 2006;44: 467–471. 10.1080/13693780500543238 16882614

[38] Tsuneto LT, Arce-Gomez B, Petzl-Erler ML, Queiroz-Telles F. HLA-A29 and genetic susceptibility to chromoblastomycosis. J Med Vet Mycol 1989;27: 181–185. 10.1080/02681218980000241 2778577

[39] Lionakis MS, Netea MG, Holland SM. Mendelian genetics of human susceptibility to fungal infection. Cold Spring Harb Perspect Med. 2014;4(6): a019638 10.1101/cshperspect.a019638 24890837 PMC4031953

[40] Holland SM. Chronic Granulomatous Disease. Hematol Oncol Clin North Am. 2013;27: 89–99. 10.1016/j.hoc.2012.11.002 23351990 PMC3558921

[41] Maskarinec SA, Johnson MD, Perfect JR. Genetic Susceptibility to Fungal Infections: What is in the Genes? Curr Clin Microbiol Reports. 2016;3: 81–91. 10.1007/s40588-016-0037-3 PMC4988683 27547700

[42] van de Sande WWJ, Fahal A, Verbrugh H, van Belkum A. Polymorphisms in Genes Involved in Innate Immunity Predispose Toward Mycetoma Susceptibility. J Immunol. 2007;179: 3065–3074. 10.4049/jimmunol.179.5.3065 17709521

[43] Puel A, Cypowyj S, Bustamante J, Wright JF, Liu L, Lim HK, et al Chronic mucocutaneous candidiasis in humans with inborn errors of interleukin-17 immunity. Science. 2011; 332(6025): 65–68. 10.1126/science.1200439 21350122 PMC3070042

[44] van de Veerdonk FL, Plantinga TS, Hoischen A, Smeekens SP, Joosten LAB, Gilissen C, et al STAT1 Mutations in Autosomal Dominant Chronic Mucocutaneous Candidiasis. N Engl J Med. 2011;365: 54–61. 10.1056/NEJMoa1100102 21714643

[45] Anderson MS, Venanzi ES, Klein L, Chen Z, Berzins SP, Turley SJ, et al Projection of an immunological self shadow within the thymus by the aire protein. Science. 2002;298: 1395–1401. 10.1126/science.1075958 12376594

[46] Puel A, Döffinger R, Natividad A, Chrabieh M, Barcenas-Morales G, Picard C, et al Autoantibodies against IL-17A, IL-17F, and IL-22 in patients with chronic mucocutaneous candidiasis and autoimmune polyendocrine syndrome type I. J Exp Med. 2010;207: 291–297. 10.1084/jem.20091983 20123958 PMC2822614

[47] van de Sande WWJ. Global Burden of Human Mycetoma: A Systematic Review and Meta-analysis. PLoS Negl Trop Dis. 2013;7: e2550 10.1371/journal.pntd.0002550 24244780 PMC3820768

[48] Sumangala B, Sahana Shetty NS, Kala B, Kavya M. Mycetoma Foot caused by Fusarium solani in a Young Boy from Karnataka, India. Int. J. Curr. Microbiol. App. Sci. 2016;5: 307–310.

[49] Taylor PR, Leal SM, Sun Y, Pearlman E. Aspergillus and Fusarium Corneal Infections Are Regulated by Th17 Cells and IL-17–Producing Neutrophils. J Immunol. 2014;192: 3319–3327. 10.4049/jimmunol.1302235 24591369 PMC4020181

[50] Siddig EE, Mohammed Edris AM, Bakhiet SM, van de Sande WWJ, Fahal AH. Interleukin-17 and matrix metalloprotease-9 expression in the mycetoma granuloma. PLoS Negl Trop Dis. 2019;13: e0007351 10.1371/journal.pntd.0007351 31295246 PMC6622479

[51] Maziarz EK, Perfect JR. Cryptococcosis. Infect Dis Clin North Am. 2016;30: 179–206. 10.1016/j.idc.2015.10.006 26897067 PMC5808417

[52] Kumar V, Cheng S-C, Johnson MD, Smeekens SP, Wojtowicz A, Giamarellos-Bourboulis E, et al Immunochip SNP array identifies novel genetic variants conferring susceptibility to candidaemia. Nat Commun. 2014;5: 4675 10.1038/ncomms5675 25197941 PMC5142444

[53] Cunha C, Aversa F, Romani L, Carvalho A. Human Genetic Susceptibility to Invasive Aspergillosis. PLoS Pathog. 2013;9: e1003434 10.1371/journal.ppat.1003434 23950708 PMC3738488

[54] Latgé JP. The pathobiology of Aspergillus fumigatus. Trends Microbiol. 2001;9: 382–389. 10.1016/s0966-842x(01)02104-7 11514221

[55] Kesh S, Mensah NY, Peterlongo P, Jaffe D, Hsu K, VAN DEN Brink M, et al TLR1 and TLR6 polymorphisms are associated with susceptibility to invasive aspergillosis after allogeneic stem cell transplantation. Ann N Y Acad Sci. 2005;1062: 95–103. 10.1196/annals.1358.012 16461792

[56] Carvalho A, Pasqualotto AC, Pitzurra L, Romani L, Denning DW, Rodrigues F. Polymorphisms in Toll‐Like Receptor Genes and Susceptibility to Pulmonary Aspergillosis. J Infect Dis. 2008;197: 618–621. 10.1086/526500 18275280

[57] Carvalho A, De Luca A, Bozza S, Cunha C, D’Angelo C, Moretti S, et al TLR3 essentially promotes protective class I-restricted memory CD8 T-cell responses to Aspergillus fumigatus in hematopoietic transplanted patients. Blood. American Society of Hematology; 2012;119: 967–977. 10.1182/blood-2011-06-362582 22147891

[58] Loetscher M, Gerber B, Loetscher P, Jones S a, Piali L, Clark-Lewis I, et al Chemokine receptor specific for IP10 and mig: structure, function, and expression in activated T-lymphocytes. J Exp Med. 1996;184: 963–9. 10.1084/jem.184.3.963 9064356 PMC2192763

[59] Mezger M, Steffens M, Beyer M, Manger C, Eberle J, Toliat MR, et al Polymorphisms in the chemokine (C-X-C motif) ligand 10 are associated with invasive aspergillosis after allogeneic stem-cell transplantation and influence CXCL10 epression in monocyte-derived dendritic cells. Blood. 2008;111: 534–536. 10.1182/blood-2007-05-090928 17957030

[60] Lambourne J, Agranoff D, Herbrecht R, Troke PF, Buchbinder A, Willis F, et al Association of Mannose‐Binding Lectin Deficiency with Acute Invasive Aspergillosis in Immunocompromised Patients. Clin Infect Dis. 2009;49: 1486–1491. 10.1086/644619 19827955

[61] Cunha C, Aversa F, Lacerda JF, Busca A, Kurzai O, Grube M, et al Genetic PTX3 Deficiency and Aspergillosis in Stem-Cell Transplantation. N Engl J Med. 2014;370: 421–432. 10.1056/NEJMoa1211161 24476432

[62] Zaas AK, Liao G, Chien JW, Weinberg C, Shore D, Giles SS, et al Plasminogen alleles influence susceptibility to invasive aspergillosis. PLoS Genet. 2008;4: e1000101 10.1371/journal.pgen.1000101 18566672 PMC2423485

[63] Förstermann U, Sessa WC. Nitric oxide synthases: Regulation and function. Eur Heart J. Oxford University Press; 2012;33: 829–37, 837a-837d. 10.1093/eurheartj/ehr304 21890489 PMC3345541

[64] Mercereau‐Puijalon O, May J, Mordmüller B, Perkins DJ, Alpers M, Weinberg JB, et al Nitric Oxide Synthase 2 Lambaréné (G‐954C), Increased Nitric Oxide Production, and Protection against Malaria. J Infect Dis. 2002;184: 330–336. 10.1086/322037 11443559

[65] Dovi J V, He L-K, DiPietro LA. Accelerated wound closure in neutrophil-depleted mice. J Leukoc Biol. 2003;73: 448–55. 10.1189/jlb.0802406 12660219

[66] Stenger S, Donhauser N, Thüring H, Röllinghoff M, Bogdan C. Reactivation of latent leishmaniasis by inhibition of inducible nitric oxide synthase. J Exp Med. 1996;183: 1501–14. 10.1084/jem.183.4.1501 8666908 PMC2192515

[67] Mangalam AK, Taneja V, David CS. HLA Class II Molecules Influence Susceptibility versus Protection in Inflammatory Diseases by Determining the Cytokine Profile. J Immunol. 2013;190: 513–519. 10.4049/jimmunol.1201891 23293357 PMC3545203

[68] Dubaniewicz A, Lewko B, Moszkowska G, Zamorska B, Stepinski J. Molecular subtypes of the HLA-DR antigens in pulmonary tuberculosis. Int J Infect Dis. 2000;4: 129–133. 10.1016/s1201-9712(00)90073-0 11179915

[69] Hilda Carrillo-Meléndez, Esteban Ortega-Hernández, Granados Julio, Arroyo Sara, Rodrigo Barquera RA. Role of HLA-DR Alleles to Increase Genetic Susceptibility to Onychomycosis in Nail Psoriasis. Ski Appendage Disord. 2016;2: 22–25. 10.1159/000446444 PMC5096265 27843918

[70] Marques RE, Guabiraba R, Russo RC, Teixeira MM. Targeting CCL5 in inflammation. Expert Opin Ther Targets. England; 2013;17: 1439–1460. 10.1517/14728222.2013.837886 24090198 PMC7103722

[71] Antachopoulos C, Roilides E. Cytokines and fungal infections. Br J Haematol. 2005;129(5): 583–596. 10.1111/j.1365-2141.2005.05498.x 15916680

[72] Geneugelijk K, Kloezen W, Fahal AH, van de Sande WWJ. Active Matrix Metalloprotease-9 Is Associated with the Collagen Capsule Surrounding the Madurella mycetomatis Grain in Mycetoma. PLoS Negl Trop Dis. 2014;8: e2754 10.1371/journal.pntd.0002754 24675764 PMC3967957

[73] Ahmed AO, van Leeuwen W, Fahal A, van de Sande W, Verbrugh H, van Belkum A. Mycetoma caused by Madurella mycetomatis: a neglected infectious burden. Lancet Infect Dis. 2004;4: 566–74. 10.1016/S1473-3099(04)01131-4 15336224

[74] Verwer PEB, Notenboom CC, Eadie K, Fahal AH, Verbrugh HA, van de Sande WWJ. A Polymorphism in the Chitotriosidase Gene Associated with Risk of Mycetoma Due to Madurella mycetomatis Mycetoma–A Retrospective Study. PLoS Negl Trop Dis. 2015;9: e0004061 10.1371/journal.pntd.0004061 26332238 PMC4558086

[75] Boot RG, Renkema GH, Verhock M, Strijland A, Bliek J, De Meulemeester TMAMO, et al The human chitotriosidase gene—Nature of inherited enzyme deficiency. J Biol Chem. 1998;273: 25680–25685. 10.1074/jbc.273.40.25680 9748235

[76] Seibold MA, Donnelly S, Solon M, Innes A, Woodruff PG, Boot RG, et al Chitotriosidase is the primary active chitinase in the human lung and is modulated by genotype and smoking habit. J Allergy Clin Immunol. 2008;122: 944–950.e3. 10.1016/j.jaci.2008.08.023 18845328 PMC2666777

[77] van de Sande WW, Fahal A, Tavakol M, van Belkum A. Polymorphisms in catechol-O-methyltransferase and cytochrome p450 subfamily 19 genes predispose towards Madurella mycetomatis- induced mycetoma susceptibility. Med Mycol 2010; 48: 959–968. 10.3109/13693781003636680 20184498

[78] Lek M, Karczewski KJ, Minikel E V., Samocha KE, Banks E, Fennell T, et al Analysis of protein-coding genetic variation in 60,706 humans. Nature. 2016;536: 285–91. 10.1038/nature19057 27535533 PMC5018207

[79] Newport M. The Contribution of Host Genetics to TB Disease. Curr Respir Med Rev. 2013;9: 156–162.

[80] Vera-Cabrera L, Ortiz-Lopez R, Elizondo-Gonzalez R, Perez-Maya AA, Ocampo-Candiani J. Complete genome sequence of Nocardia brasiliensis HUJEG-1. J Bacteriol. 2012;194: 2761–2762. 10.1128/JB.00210-12 22535940 PMC3347167

[81] Kirby R, Sangal V, Tucker NP, Zakrzewska-Czerwińska J, Wierzbicka K, Herron PR, et al Draft genome sequence of the human pathogen Streptomyces somaliensis, a significant cause of actinomycetoma. J Bacteriol. 2012;194: 3544–3545. 10.1128/JB.00534-12 22689234 PMC3434723

[82] Vera-Cabrera L, Ortiz-Lopez R, Elizondo-Gonzalez R, Campos-Rivera MP, Gallardo-Rocha A, Molina-Torres CA, et al Draft Genome Sequence of Actinomadura madurae LIID-AJ290, Isolated from a Human Mycetoma Case. Genome Announc. American Society for Microbiology (ASM); 2014;2: e00201–14. 10.1128/genomeA.00201-14 24675856 PMC3968334

[83] Shaw JJ, Spakowicz DJ, Dalal RS, Davis JH, Lehr NA, Dunican BF, et al Biosynthesis and genomic analysis of medium-chain hydrocarbon production by the endophytic fungal isolate Nigrograna mackinnonii E5202H. Appl Microbiol Biotechnol. 2015;99: 3715–3728. 10.1007/s00253-014-6206-5 25672844 PMC4667366

[84] Smit S, Derks MFL, Bervoets S, Fahal A, van Leeuwen W, van Belkum A, et al Genome Sequence of Madurella mycetomatis mm55, Isolated from a Human Mycetoma Case in Sudan. Genome Announc. 2016;4: e00418–16. 10.1128/genomeA.00418-16 27231361 PMC4882942

[85] Sagoo GS, Little J, Higgins JPT. Systematic Reviews of Genetic Association Studies. PLoS Med. 2009;6: e1000028. 10.1371/journal.pmed.1000028 PMC2650724 19260758

[86] Gorroochurn P, Hodge SE, Heiman GA, Durner M, Greenberg DA. Non-replication of association studies: “Pseudo-failures” to replicate? Genet Med. 2007;9: 325–331. 10.1097/gim.0b013e3180676d79 17575498

